# Hibernoma: an uncommon cause of a pleural mass

**DOI:** 10.1590/S1806-37132015000100015

**Published:** 2015

**Authors:** Edson Marchiori, Gláucia Zanetti, Bruno Hochhegger

**Affiliations:** Federal University of Rio de Janeiro, Rio de Janeiro, Brazil, Fluminense Federal University, Niterói, Brazil; and Associate Professor of Radiology, Federal University of Rio de Janeiro, Rio de Janeiro, Brazil; Petrópolis School of Medicine, Petrópolis, Brazil, Graduate Program in Radiology, Federal University of Rio de Janeiro, Rio de Janeiro, Brazil; and Professor of Clinical Medicine, Petrópolis School of Medicine, Petrópolis, Brazil; Federal University of Health Sciences of Porto Alegre, Porto Alegre, Brazil, Santa Casa Hospital Complex in Porto Alegre; and Professor of Radiology, Federal University of Health Sciences of Porto Alegre, Porto Alegre, Brazil

**Figure 1 -:**
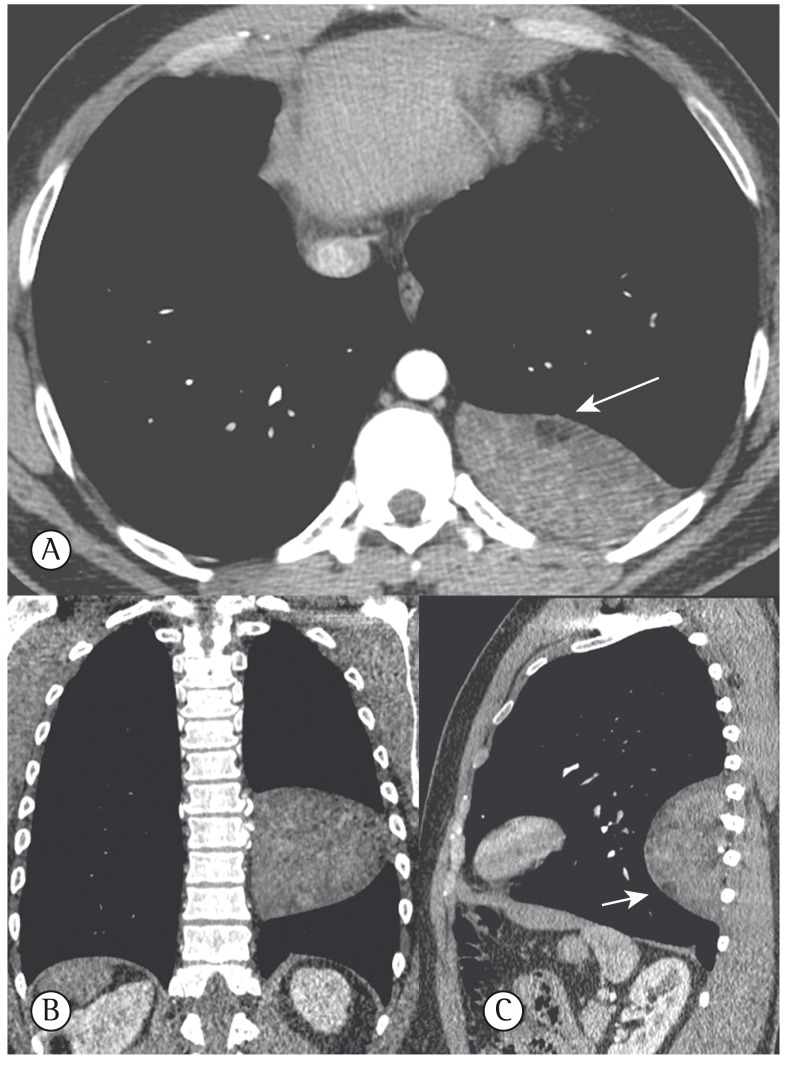
Chest CT reconstructions in the axial, coronal, and sagittal planes (A, B, and C, respectively), showing a heterogeneous pleural mass with areas of low-attenuation (fatty tissue, arrows) in the left lower hemithorax.

**Figure 2 -:**
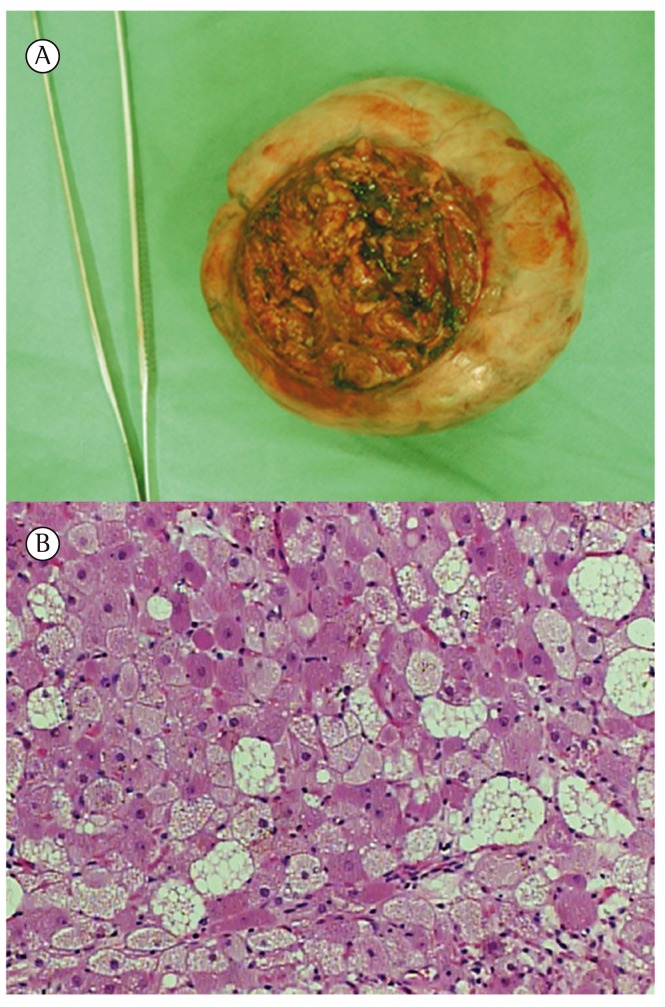
Photograph of the gross specimen (A), demonstrating a well-circumscribed, encapsulated, soft, brown-to-yellow mass measuring 10 × 9 × 5 cm. Below, the photomicrograph (B) shows that, histologically, the tumor consisted of two tumor cell types: cells with granular intense eosinophilic cytoplasm; and clear multivacuolated cells filled with lipid droplets, with no evidence of cellular atypia or mitosis (hematoxylin and eosin staining; magnification, ×200).

To the Editor:

Here, we report the case of a 37-year-old asymptomatic male patient who was referred because of abnormalities seen on a routine chest X-ray. The physical examination findings and laboratory test results were normal. A new chest X-ray revealed a large opacity in the left lower hemithorax. Chest CT revealed a heterogeneous pleural mass in the left lower hemithorax (Fig. 1). The mass appeared to be an extrapulmonary lesion arising from the chest wall. There were no calcifications. Complete surgical excision was performed. The gross specimen showed a well-circumscribed, encapsulated, soft, brown-to-yellow mass, measuring 10 × 9 × 5 cm (Fig. 2A). The microscopic findings were diagnostic of a hibernoma (Fig. 2B). At this writing, the patient remains asymptomatic and subsequent follow-up evaluations have been unremarkable.

Hibernomas are rare benign tumors that take their name from their histological similarity to the brown fat of hibernating animals, but also seen in the human fetus and to a diminishing degree with age in adults.^(^ 1 ^-^ 3 ^)^ The distribution of this tumor follows the sites of persistence of brown fat. The most common sites are the thigh, shoulder, back, neck, thorax, upper extremity, abdomen, and retroperitoneum. Intrathoracic locations include the mediastinum and the pericardium. Tumors involving the pleura are extremely uncommon. In most cases, a hibernoma manifests as a painless mass and is an incidental finding on physical examination or imaging. Although these tumors are always benign, they tend to grow to large sizes and symptoms can arise from the compression of adjacent structures. In individuals with a hibernoma, significant weight loss has been described and is attributed to excessive thermogenesis of the tumor tissue responsible for the catabolism of circulating lipids and carbohydrates into thermal energy.^(^ 4 ^)^ Complete surgical excision is the treatment of choice, and the postoperative prognosis is excellent. There have been no reports of recurrence or metastatic disease in hibernoma patients.^(^ 1 ^,^ 2 ^)^ The gross specimen typically shows a well-encapsulated, firm, tan or brown tumor. Microscopy reveals univacuolated or multivacuolated fat cells with small, central nucleoli.^(^ 1 ^)^

On CT scans, a hibernoma usually presents as a heterogenous low-attenuation mass (with regions of fat and soft tissue attenuation); on T1- and T2-weighted magnetic resonance imaging, it is seen as a hyperintense, heterogeneous mass. In imaging studies, the main differential diagnoses are lipoma and liposarcoma. Because of the similarity of their fat content, hibernomas and lipomas have comparable signal characteristics on magnetic resonance imaging and CT scans. Although hibernomas are more heterogeneous due to their different composition, in terms of their fibrous and vascular elements, histopathological analysis is always necessary in order to make an accurate diagnosis.^(^ 1 ^,^ 2 ^)^
